# The Relationship between VEGFC Gene Polymorphisms and Autoimmune Thyroiditis

**DOI:** 10.1155/2022/2603519

**Published:** 2022-07-12

**Authors:** Chaoqun Gao, Jie Zhu, Qiu Qin, Xiaorong Yang, Yanfei Jiang, Jinan Zhang

**Affiliations:** ^1^Graduate School of Shanghai University of Traditional Chinese Medicine, Shanghai 201203, China; ^2^Department of Endocrinology & Rheumatology, Shanghai University of Medicine & Health Sciences Affiliated Zhoupu Hospital, Shanghai 201318, China; ^3^Shanghai University of Traditional Chinese Medicine, Shanghai 201203, China

## Abstract

**Background:**

Autoimmune thyroid diseases (AITDs), representative autoimmune diseases, mainly consist of Graves' disease (GD) and Hashimoto's thyroiditis (HT). In this passage, we investigated the association between vascular endothelial growth factor C (VEGFC) gene polymorphisms and AITDs.

**Methods:**

A total of 1084 patients with AITDs and 794 healthy controls were tested for VEGFC gene genotypes in four single nucleotide polymorphisms (SNPs) by high-throughput sequencing, and the correlation between VEGFC gene polymorphisms and AITDs was statistically analyzed.

**Results:**

The genotype distribution of rs3775194 was statistically associated with AITDs compared with the control group. Rs3775194 was associated with AITDs under the overdominant model, both before and after adjusting for confounding factors, while the other three SNPs were not associated with GD and HT. There was a prominent discrepancy between male healthy controls and male AITD patients under overdominant model in rs3775194 and the recessive model in rs11947611. The genotype distribution of rs3775194 was statistically related to male HT.

**Conclusion:**

These results reveal the correlation between VEGFC mutation and AITD susceptibility.

## 1. Introduction

AITDs are thyroid-specific autoimmune diseases caused by the disorder of autoimmune mechanisms, among which GD and HT are the major subtypes [1]. GD and HT have different clinical manifestations and pathophysiological characteristics. GD shows elevated TSH receptor stimulating antibody (TRAb), accompanied with lymphocyte infiltration in the thyroid gland and hypertrophy of thyroid follicular epithelial cells. HT shows interstitial fibrous tissue hyperplasia and destruction of thyroid follicles, accompanied with elevated anti-thyroglobulin antibody (TgAb) and thyroid peroxidase antibody (TPOAb). According to related research, AITDs affect about 5% of the total population and are more popular in women than in men [2]. The pathogenesis of AITDs has not been clearly studied. Genetics, immunity, and environment may be involved in the occurrence and development of AITDs [3].

Vascular endothelial growth factor (VEGF) mainly involves in neovascular diseases such as malignant tumors and plays a role in increasing vascular permeability and mediating inflammation. It is affiliated with the platelet-derived growth factor family. Angiogenesis plays a role in a variety of autoimmune inflammatory diseases, including rheumatoid arthritis [4], systemic lupus erythematosus [5], and systemic sclerosis [6]. Inhibition of angiogenesis may be a promising treatment of these diseases. VEGFs are angiogenic factors which contain five members in mammals: VEGFA, VEGFB, VEGFC, VEGFD, and placental growth factor (PGF). VEGFC is encoded in humans by the VEGFC gene, which is located on chromosome 4q34. VEGFC is a ligand of VEGF-R3 and VEGF-R2, but it exerts effects mainly through VEGF-R3 [7]. It is widely believed that VEGFC plays a major role in lymphangiogenesis and promotes the survival, growth, and migration of lymphatic endothelial cells (LECs) through its receptor VEGFR-3 [8]. We hypothesized that VEGFC gene is related to autoimmune thyroid diseases. Therefore, we investigated the association between VEGFC gene polymorphisms and AITD risk using a case-control approach.

## 2. Methods

### 2.1. Recruitment of the Participants

We recruited 1084 patients with AITD and 794 healthy controls from the Han Chinese population. AITD patients consisted of 256 men and 828 women. The normal healthy group was made up of 317 men and 477 women. To eliminate sampling error, all AITD patients were randomly recruited from the outpatient department of Shanghai Zhoupu Hospital, and the normal controls were consecutively enrolled from physical examination center of the same hospital. All AITD patients and normal controls were free of other autoimmune and inflammatory diseases. The participants were all from Shanghai, China. The study was permitted by the Ethics Committee of Shanghai University of Medicine & Health Sciences, and all the subjects in the study provided written informed consent.

In the AITD patients, there were 667 patients with GD (191 males and 476 females) and 417 patients with HT (65 males and 352 females). Patients with GD in the study were required to meet the following criteria: typical symptoms of hyperthyroidism, biochemical tests of hyperthyroidism, and positive TRAb [9]. Positive TPOAb or TgAb, and ultrasonographic findings of diffuse thyroid changes are the basis for the diagnosis of HT. TRAb, TgAb, TPOAb, and other serological parameters were detected by immunochemiluminescence kit (Roche Company, Switzerland) with high quality.

In order to explore the correlation between different clinical manifestations and genetic background, the relationship between SNP and various clinical subtypes was elaborated. In this study, the clinical manifestations such as Graves' ophthalmopathy (GO) in GD patients and hypothyroidism in HT patients were included.  Table 1 summarizes the clinical characteristics of all the subjects. GO, also known as thyroid-related eye disease, is characterized by exophthalmos, excessive tearing, painful eye movement, and diplopia. We can make a diagnosis based on the criteria in Williams Textbook of Endocrinology.

### 2.2. Isolation and Extraction of DNA Samples

Two-milliliter peripheral venous blood was collected from each individual. Patients' DNA were extracted using Relax Gene Blood DNA System (Tiangen Biotech Co., Ltd., Beijing, China). The quality of genomic DNA was evaluated by Nano Drop 2000 spectrophotometer (Thermo Scientific Company, Waltham, USA) according to the manufacturer's guidelines.

### 2.3. SNP Selection and Genotyping

This study examined the four SNPs of VEGFC (i.e., rs7664413, rs11947611, rs2046463, and rs3775194). According to previous reports, these loci are associated with several autoimmune diseases. We hypothesized that these loci are also associated with AITD susceptibility. The four SNPs need to conform to Hardy-Weinberg equilibrium (HWE) with *P* value greater than 0.05 and minor allele frequency (MAF) > 0.05. Genotypes of DNA samples were tested with high-throughput SNP sequencing. In simple terms, the DNA sample was amplified in 10 microliter volume. The temperature condition was set to 95 °C for 15 minutes, followed by five cycles of 94 °C for 30s, 60 °C for 4 minutes, and 72 °C for 30 seconds and 10 cycles 94 °C for 30s, 60 °C for 1 minute, and 72 °C for 30s. The primer sequence we used is GAGTTTCTGTCTAGTTCTTTGTGG and GGAAAACATACAAAAGGAAGATGC for rs7664413. For rs11947611, the primer sequences are AAACCTTGGCTTCTAACAATCTTC and CCTGAAAACATAAACCAAAAAGCC; TGTTTACGATACTCTCACTTTTGG and GGCCATGTAAAGAATAGTAGAACC are the primer sequences of rs2046463. TCACAGCTTAAGACTGAAATCAAC and ATTCTGTGACGATGTACTATAGGG are the primer sequences of rs3775194, respectively. In order to ensure the accuracy of the genotyping process, PCR was performed twice each time and negative control was performed using water as template.

### 2.4. Statistical Analysis

Statistical analysis included OR values, *P* values and 95% confidence intervals. We used SPSS (22.0, IBM, Chicago, USA) for our calculations. Measurement data are expressed as the mean plus or minus standard errors. The alleles and genotype frequency of SNP were analyzed by Chi-square test. By adjusting potential confounding factors such as thyroid function and gender, *P* and OR values before and after adjustment were calculated by multifactor logistic regression analysis to find out meaningful results. The correlation between VEGFC gene polymorphisms and AITD was further calculated under allele model, overdominant model, and recessive model. Linkage analysis was calculated using Haploview 4.2 software (Broad Institute, Cambridge, USA) with *p* < 0.05 deemed as positive.

## 3. Results

Table 2 shows the distribution of alleles and genotypes of VEGFC loci in AITD, GD, HT, and controls. No meaningful conclusions were found in the alleles and genotypes distributions of these four SNPs in GD and HT patients between cases and those in the control group. However, the genotype distribution of rs3775194 in AITD subjects (CC 1.1%, GG 83.6%, and CG 15.3%) was statistically different from that in the normal group (CC 0.5%, GG 80.1%, CG 19.4%) (*P* = 0.029). To further analyse the potential association between VEGFC gene polymorphisms and AITDs, we performed an analysis of four models before and after adjusting for confounding factors (age, sex). As demonstrated in Table 3, rs3775194 locus was strongly associated with AITD in overdominant model, both before and after adjusting for confounders (*P* = 0.021 and 0.024, respectively). No positive results were found in our study comparing GD and HT with normal controls (data are not presented).

To further investigate whether VEGFC loci are associated with AITD in different genders, as presented in Table 4, we found that rs11947611 was associated with male AITD patients in a recessive model, both before and after adjustment for confounders (*P* = 0.035 and 0.024, respectively). Rs3775194 was associated with male AITD patients in an overdominant model with *P* values of 0.017 and 0.023 before and after adjustment for confounding factors.  Table 5 shows the genotypes and allele frequencies of these four loci in different sex subgroups of HT patients. The genotype distribution of rs3775194 in male HT patients (CC 1.5%, GG 87.7%, and CG 10.8%) was significantly different from that in male control group (GG 78.2% and CG21.8%) (*P* = 0.048), but there were no positive results in allele frequency between the two groups. We have not yet found an association between VEGFC loci and female AITD patients (Table 6). No distinguish difference was found between the female HT and the control group in four loci.

We further analyzed the correlation between four loci of VEGFC and some clinical phenotypes. It can be seen from Table 7 that there was no correlation between four polymorphisms of VEGFC and susceptibility to Graves' ophthalmopathy (GO).  Table 8 shows that the distribution of these loci was not associated with hypothyroidism in HT patients, either.

**Figure 1:**
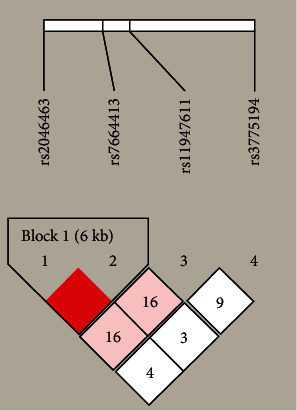
VEGFC linkage disequilibrium (LD) block in the Haploview 4.2.

Haploview software showed that rs2046463 and rs7664413 formed only one linkage disequilibrium (LD) region and three main haplotypes: CA, TG, and CG. However, we did not detect an association of VEGFC haplotypes CA, TG, and CG (Figure 1) with susceptibility to AITD, GD, and HT.

## 4. Discussion

Epidemiological studies have confirmed that genetic factors play an important role in AITDs, but the known genes related to AITDs cannot fully explain the role of genetic factors in AITDs. In this study, we explored the relationship between VEGFC gene polymorphisms and AITDs using allele, dominant, recessive, and overdominant models as well as different subgroups of AITD. We found that the rs3775194 locus was associated with AITD patients, but not with GD or HT patients. We further found that rs3775194 was associated with male AITD patients under the overdominant model and rs3775194 was associated with the genotype distribution of male HT patients. Rs11947611 is associated with male AITD patients under the recessive model.

VEGF is a functional glycoprotein with high biological activity. It is also called vascular permeability factor due to its strong ability to promote the differentiation and proliferation of vascular endothelial cells. VEGF gene is involved in the occurrence and development of diabetic retinopathy and cancer [10, 11]. A study of 1 919 diabetic patients with gene polymorphisms found that three SNPs (rs17697419, rs17697515, and rs2333526) of VEGFC are associated with diabetic retinopathy. Rs17697515 is also specifically associated with diabetic macular edema in T2DM patients [12]. VEGFC gene also plays an important role in the development of various autoimmune diseases such as rheumatoid arthritis [4]. VEGF-C/sVEGFR-3 ratio is significantly lower in patients with Behcet's disease than in the control group and is correlated with the course of the disease [13]. In addition, serum VEGFC values are higher in adult-onset Still's disease, which may be a marker of disease activity [14]. On the other hand, VEGFC levels are low in patients with systemic sclerosis, and VEGFC may be a useful indicator for early prediction of pulmonary arterial hypertension in those patients [15]. Furthermore, VEGFC aggravates intestinal inflammation in mice with experimental colitis and is associated with inflammatory lymphatic formation [16]. Similarly, VEGFCs are highly expressed in salivary duct epithelial cells in patients with primary Sjogren's syndrome, and lymphangiogenesis is active in this syndrome [17]. Based on the facts above, it is reasonable to suspect that VEGFC is involved in AITD. Therefore, this study aimed to investigate the relationship between VEGFC gene polymorphisms and AITDs.

VEGFC gene is located on chromosome 4q34.3 and has many SNPs, including rs7664413, rs11947611, rs2046463, and rs3775194. All four of them are located in the intron region. As we discussed previously, only the rs3775194 genotype distribution of the four SNPs was associated with AITD compared with the control group. Neither the allele nor genotype of four SNPs was involved in GD subgroup. Since AITDs are sex-specific diseases, we found significant differences in the genotype distribution of rs3775194 in male HT patients compared with controls. In addition, rs3775194 was significantly associated with AITD and male AITD patients under the overdominant model. Moreover, rs11947611 was associated with male AITD under the recessive model. We did not find any significant conclusions in the four SNPs of VEGFC comparing GO in GD and hypothyroidism in HT with corresponding controls. The lack of significant positive results might be due to the differences in the influence of environmental and genetic factors on the Han population and the limited number of sampled individuals. Further large population studies warrant further study.

In genomic DNA, changes in a single base may affect the amino acid sequence and ultimately affects susceptibility to diseases [18]. The phenotype is the result of environmental, genetic and other factors. Although these loci, such as rs3775194, are localized to noncoding regions, they may influence disease occurrence by regulating gene structure or expression [19]. VEGFC gene is highly expressed in thyroid tissue [20], whereas in patients with AITD, there is an increase in lymphocyte infiltration in thyroid tissue, which suggests lymphatic hyperplasia in thyroid tissue.

Haplotype analysis is a more powerful way to prove that a gene is associated with a disease. We found a strong linkage disequilibrium in 2 SNPs between patients and controls. However, subsequent study showed no association between haplotypes and AITD, HT, or GD.

In conclusion, rs3775194 locus was associated with AITD, male AITD, and male HT patients, and rs11947611 was associated with male AITD patients. VEGFC loci is related to the immune system and may be a risk factor for AITDs.

## Figures and Tables

**Table 1:** Clinical features and demographic statistics of AITD patients and controls.

Items	AITD (%)	GD (%)	HT (%)	Controls (%)
Number	1084	667	417	794
Age	41.7 ± 14.3	41 ± 14.6	42.8 ± 13.8	38.9 ± 10.5
Sex				
Females	828 (76.4)	476 (71.4)	352 (84.4)	477 (60.1)
Males	256 (23.6)	191 (28.6)	65 (15.6)	317 (39.9)
Ophthalmopathy (+)	/	99 (15.8)	/	/
Hypothyroidism (+)	/	/	174 (42.5)	/

**Table 2:** Associations of rs7664413, rs11947611, rs2046463, and rs3775194 in VEGFC gene with AITD, GD, and HT.

		AITD *n* (%)	NC *n* (%)	P value AITD vs NC	GD *n* (%)	P value GD vs NC	HT *n* (%)	P value HT vs NC
rs7664413	CC	495 (45.7)	350 (44.1)	0.652	293 (43.9)	0.364	202 (48.4)	0.238
	TT	122 (11.3)	85 (10.7)		87 (13.0)		35 (8.4)	
	TC	467 (43.1)	359 (45.2)		288 (43.1)		180 (43.2)	
	C	1457 (67.2)	1059 (66.7)	0.739	874 (65.4)	0.470	584 (70.0)	0.095
	T	711 (32.8)	529 (33.3)		462 (34.6)		250 (30.0)	
rs11947611	AA	410 (37.8)	318 (40.1)	0.527	251 (37.6)	0.534	159 (38.1)	0.765
	GG	167 (15.4)	111 (14.0)		104 (15.6)		63 (15.1)	
	AG	507 (46.8)	365 (46.0)		313 (46.9)		195 (46.8)	
	A	1327 (61.2)	1001 (63.0)	0.255	815 (61.0)	0.259	513 (61.5)	0.461
	G	841 (38.8)	587 (37.0)	0.600	521 (39.0)		321 (38.5)	
rs2046463	AA	492 (45.4)	347 (43.7)		291 (43.6)	0.315	201 (48.2)	0.224
	GG	123 (11.3)	85 (10.7)		88 (13.2)		35 (8.4)	
	AG	469 (43.3)	362 (45.6)		289 (43.3)		181 (43.4)	
	A	1453 (67.0)	1056 (66.5)	0.737	871 (65.2)	0.459	583 (69.9)	0.089
	G	715 (33.0)	532 (33.5)		465 (34.8)		251 (30.1)	
rs3775194	CC	12 (1.1)	4 (0.5)	0.029	8 (1.2)	0.092	4 (1.0)	0.052
	GG	906 (83.6)	636 (80.1)		553 (82.8)		354 (84.9)	
	CG	166 (15.3)	154 (19.4)		107 (16.0)		59 (14.1)	
	C	190 (8.8)	162 (10.2)	0.135	123 (9.1)	0.310	67 (8.0)	0.083
	G	1978 (91.2)	1426 (89.8)		1213 (90.9)		767 (92.0)	

**Table 3:** Associations of four polymorphisms models in VEGFC with AITD before and after adjusting for confounders.

Comparison models	Unadjusted estimates		Adjusted estimates	
	OR (95% CI)	*P* value	OR(95% CI)	*P* value
rs7664413				
Allele model	0.98 (0.85-1.12)	0.74	0.97 (0.84-1.12)	0.69
Dominant model	0.94 (0.78-1.13)	0.50	0.93 (0.77-1.12)	0.45
Recessive model	1.06 (0.79-1.42)	0.71	1.06 (0.78-1.43)	0.72
Overdominant model	0.92 (0.76-1.10)	0.36	0.91 (0.75-1.10)	0.32
rs11947611				
Allele model	1.08 (0.95-1.23)	0.26	1.06 (0.92-1.21)	0.43
Dominant model	1.10 (0.91-1.32)	0.33	1.07 (0.89-1.30)	0.47
Recessive model	1.12 (0.86-1.45)	0.39	1.08 (0.83-1.40)	0.59
Overdominant model	1.03 (0.86-1.24)	0.73	1.03 (0.86-1.24)	0.74
rs2046463				
Allele model	0.98 (0.85-1.12)	0.74	0.97 (0.84-1.12)	0.69
Dominant model	0.93 (0.78-1.12)	0.47	0.93 (0.77-1.12)	0.43
Recessive model	1.07 (0.80-1.43)	0.66	1.06 (0.79-1.43)	0.69
Overdominant model	0.91 (0.76-1.09)	0.32	0.90 (0.75-1.09)	0.29
rs3775194				
Allele model	0.85 (0.68-1.05)	0.14	0.83 (0.66-1.05)	0.12
Dominant model	0.79 (0.62-1.00)	0.05	0.79 (0.62-1.00)	0.05
Recessive model	2.21 (0.71-6.88)	0.15	1.91 (0.60-6.08)	0.25
Overdominant model	0.75 (0.59-0.96)	0.02	0.75 (0.59-0.96)	0.02

**Table 4:** Associations of four polymorphisms models in VEGFC with male AITD before and after adjusting for confounders.

Comparison models	Unadjusted estimates		Adjusted estimates	
	OR (95% CI)	*P* value	OR (95% CI)	*P* value
rs7664413				
Allele model	1.07 (0.83-1.37)	0.60	1.05 (0.82-1.34)	0.72
Dominant model	1.01 (0.72-1.40)	0.96	0.98 (0.70-1.37)	0.90
Recessive model	1.32 (0.78-2.23)	0.30	1.30 (0.77-2.21)	0.33
Overdominant model	0.90 (0.65-1.26)	0.54	0.88 (0.63-1.23)	0.46
rs11947611				
Allele model	1.26 (0.98-1.61)	0.07	1.25 (0.97-1.60)	0.08
Dominant model	1.20 (0.86-1.69)	0.28	1.16 (0.82-1.63)	0.40
Recessive model	1.71 (1.04-2.82)	0.04	1.79 (1.08-2.98)	0.02
Overdominant model	0.95 (0.68-1.32)	0.74	0.89 (0.64-1.24)	0.49
rs2046463				
Allele model	1.05 (0.82-1.35)	0.68	1.03 (0.81-1.33)	0.79
Dominant model	0.98 (0.71-1.37)	0.92	0.96 (0.68-1.34)	0.79
Recessive model	1.32 (0.78-2.23)	0.30	1.30 (0.77-2.21)	0.33
Overdominant model	0.88 (0.63-1.23)	0.45	0.86 (0.62-1.20)	0.38
rs3775194				
Allele model	0.72 (0.48-1.09)	0.12	0.74 (0.49-1.12)	0.15
Dominant model	0.65 (0.42-1.00)	0.05	0.66 (0.43-1.02)	0.06
Recessive model	——	0.03	——	0.02
Overdominant model	0.59 (0.38-0.92)	0.02	0.60 (0.38-0.94)	0.02

**Table 5:** Allele and genotype distributions of VEGF loci in subgroups of HT patients.

		Female controls *n*(%)	Female HT *n*(%)	Unadjust/adjust *P* value		Male controls *n*(%)	Male HT *n*(%)	Unadjust/adjust *P* value	
rs7664413	CC	207 (43.4)	170 (48.3)	0.208		143 (45.1)	32 (49.2)	0.831	
	TT	54 (11.3)	29 (8.2)			31 (9.8)	6 (9.2)		
	TC	216 (45.2)	153 (43.5)			143 (45.1)	27 (41.5)		
	C	493 (70.0)	630 (66.0)	0.082	0.07	429 (67.7)	91 (70.0)	0.600	0.49
	T	211 (30.0)	324 (34.0)			205 (32.3)	39 (30.0)		
rs11947611	AA	185 (38.8)	136 (38.6)	0.627		133 (42.0)	23 (35.4)	0.120	
	GG	80 (16.8)	51 (14.5)			31 (9.7)	12 (18.5)		
	AG	212 (44.4)	165 (46.9)			153 (48.3)	30 (46.1)		
	A	582 (61.0)	437 (62.1)	0.660	0.69	419 (66.1)	76 (58.5)	0.091	0.19
	G	372 (39.0)	267 (37.9)			215 (33.9)	54 (41.5)		
rs2046463	AA	206 (43.2)	169 (48.0)	0.212		141 (44.5)	32 (49.2)	0.780	
	GG	54 (11.3)	29 (8.2)			31 (9.8)	6 (9.2)		
	AG	217 (45.5)	154 (43.8)			145 (45.7)	27 (41.5)		
	A	629 (65.9)	492 (69.9)	0.085	0.07	427 (67.4)	91 (70.0)	0.550	0.46
	G	325 (34.1)	212 (30.1)			207 (32.6)	39 (30.0)		
rs3775194	CC	4 (0.8)	3 (0.9)	0.506		0 (0)	1 (1.5)	0.048	
	GG	388 (81.3)	297 (84.4)			248 (78.2)	57 (87.7)		
	CG	85 (17.8)	52 (14.8)			69 (21.8)	7 (10.8)		
	C	93 (9.7)	58 (8.2)	0.290	0.23	69 (10.9)	9 (6.9)	0.140	0.17
	G	861 (90.3)	646 (91.8)			565 (89.1)	121 (93.1)		

**Table 6:** Associations of four polymorphisms models in VEGFC with female AITD before adjusting for confounders.

	OR (95% CI)	*P* value
rs7664413		
Allele model	0.96 (0.83-1.12)	0.62
Dominant model	0.93 (0.76-1.13)	0.46
Recessive model	1.02 (0.74-1.39)	0.92
Overdominant model	0.92 (0.76-1.12)	0.42
rs11947611		
Allele model	1.08 (0.93-1.24)	0.31
Dominant model	1.09 (0.90-1.34)	0.38
Recessive model	1.11 (0.85-1.47)	0.44
Overdominant model	1.03 (0.85-1.25)	0.76
rs1485766		
Allele model	1.00 (0.88-1.15)	0.95
Dominant model	1.09 (0.88-1.36)	0.43
Recessive model	0.92 (0.73-1.15)	0.48
Overdominant model	1.14 (0.94-1.39)	0.18
rs2046463		
Allele model	0.97 (0.83-1.12)	0.64
Dominant model	0.93 (0.76-1.13)	0.46
Recessive model	1.03 (0.75-1.41)	0.85
Overdominant model	0.92 (0.75-1.12)	0.39
rs3775194		
Allele model	0.86 (0.68-1.09)	0.22
Dominant model	0.81 (0.63-1.04)	0.11
Recessive model	2.17 (0.67-7.08)	0.18
Overdominant model	0.77 (0.60-1.00)	0.05

**Table 7:** Allele and genotype distributions of VEGFC polymorphisms with ophthalmopathy in GD patients.

SNPs	Without (%)	With (%)	*P* value	OR (95% CI)	
rs7664413					
CC	234 (44.4)	47 (47.5)	0.212		
TT	64 (12.1)	17 (17.2)			
TC	229 (43.5)	35 (35.4)			
C	697 (66.1)	129 (65.2)	0.790	1.044 (0.759-1.437)	
T	357 (33.9)	69 (34.8)			
rs11947611					
AA	192 (36.4)	42 (42.4)	0.433		
GG	84 (15.9)	12 (12.1)			
AG	251 (46.7)	45 (45.5)			
A	635 (60.2)	129 (65.2)	0.194	0.811 (0.59-1.113)	
G	419 (39.8)	69 (34.8)			
rs2046463					
AA	233 (44.2)	46 (46.5)	0.152		
GG	64 (12.1)	18 (18.2)			
AG	230 (43.6)	35 (35.4)			
A	696 (66.0)	127 (64.1)	0.607	1.087 (0.791-1.493)	
G	358 (34.0)	71 (35.9)			
rs3775194					
CC	4 (0.8)	3 (3.0)	0.153		
GG	440 (83.5)	80 (80.8)			
CG	83 (15.7)	16 (16.2)			
C	91 (8.6)	22 (11.1)	0.264	0.756 (0.462-1.237)	
G	963 (91.4)	176 (88.9)			

**Table 8:** Allele and genotype distributions of VEGFC polymorphisms with hypothyroidism in HT patients.

SNPs	Without (%)	With (%)	*P* value	OR (95% CI)	
rs7664413					
CC	114 (48.5)	84 (48.3)	0.981		
TT	20 (8.5)	14 (8.0)			
TC	101 (43.0)	76 (43.7)			
C	329 (70.0)	244 (70.1)	0.972	0.995 (0.735-1.346)	
T	141 (30.0)	104 (29.9)			
rs11947611					
AA	89 (37.9)	66 (37.9)	0.974		
GG	37 (15.7)	26 (14.9)			
AG	109 (46.4)	82 (47.1)			
A	287 (61.1)	214 (61.5)	0.901	0.982 (0.739-1.305)	
G	183 (38.9)	134 (38.5)			
rs2046463					
AA	113 (48.1)	84 (48.3)	0.986		
GG	20 (8.5)	14 (8.0)			
AG	102 (43.4)	76 (43.7)			
A	328 (69.8)	244 (70.1)	0.920	0.985 (0.728-1.332)	
G	142 (30.2)	104 (29.9)			
rs3775194					
CC	4 (1.7)	0 (0)	0.853		
GG	198 (84.3)	148 (85.1)			
CG	33 (14.0)	26 (14.9)			
C	41 (8.7)	26 (7.5)	0.518	1.184 (0.709-1.975)	
G	429 (91.3)	322 (92.5)			

## Data Availability

The data generated during this study are available within the article and any further information can be made available upon request to the corresponding author.
